# Effect of *You-Gui-Wan* on House Dust Mite-Induced Mouse Allergic Asthma via Regulating Amino Acid Metabolic Disorder and Gut Dysbiosis

**DOI:** 10.3390/biom11060812

**Published:** 2021-05-30

**Authors:** Wei-Hsiang Hsu, Li-Jen Lin, Chung-Kuang Lu, Shung-Te Kao, Yun-Lian Lin

**Affiliations:** 1 Department of Chinese Pharmaceutical Sciences and Chinese Medicine Resources, China Medical University, Taichung 40402, Taiwan; rabbitjim5@hotmail.com; 2 School of Chinese Medicine, College of Chinese Medicine, China Medical University, Taichung 40402, Taiwan; linlijen@mail.cmu.edu.tw (L.-J.L.); stkao@mail.cmu.edu.tw (S.-T.K.); 3 National Research Institute of Chinese Medicine, Ministry of Health and Welfare, Taipei 11221, Taiwan; cklu@nricm.edu.tw; 4 Department of Chinese Medicine, China Medical University Hospital, Taichung 40402, Taiwan; 5 Department of Pharmacy, National Taiwan University, Taipei 10050, Taiwan

**Keywords:** allergic asthma, *Dermatogoides pteronyssinus*, gut microbiota, untargeted metabolomics, *You-Gui-Wan*

## Abstract

Chinese herbal remedies have long been used for enhancing immunity and treating asthma. However, the evidence-based efficacy remains to be supported. This study aimed to explore the potential bio-signatures in allergic asthma and the effect of *You-Gui-Wan* (YGW), a traditional Chinese herbal prescription, on dust mite-induced mouse allergic asthma. Extract of *Dermatophagoides pteronyssinus* (*Der p*), a dust mite, was intratracheally administered to induce allergic asthma in mice. Serum metabolomic and 16S rRNA-based microbiome profiling were used to analyze untargeted metabolites with levels significantly changed and gut microbiota composition, respectively. Results indicated that 10 metabolites (acetylcarnitine, carnitine, hypoxanthine, tryptophan, phenylalanine, norleucine, isoleucine, betaine, methionine, and valine), mainly associated with branched-chain amino acid (BCAA) metabolism, aromatic amino acid (AAA) biosynthesis, and phenylalanine metabolism were markedly elevated after *Der p* treatment. YGW administration reversed the levels for 7 of the 10 identified metabolites, chiefly affecting BCAA metabolism. On 16S DNA sequencing, disordered *Der p*-induced gut microbiota was significantly alleviated by YGW. Multiple correlation analysis showed a good correlation between gut microbiota composition and levels of selected metabolites. Our study showed YGW administration effectively alleviated BCAA metabolic disorder and improved gut dysbiosis. This study provides support for YGW administration with benefits for allergic asthma.

## 1. Introducti

Allergic asthma, long-term airway inflammation, is prevalent worldwide and a global public health issue [1,2]; it results from gene-environment interactions and allergens [3]. Dust mites (DMs) such as *Dermatophagoides pteronyssinus* (*Der p*) and *D. farina* (*Der f*) are the major clinical allergens [4]. Most patients need long-term treatment, which has a great impact on society, economy, and medical expenditure [5]. Corticosteroids and β-agonists have been used as standard treatments for chronic asthma, which cannot be cured, but they have side effects, especially in children [6]. Allergen-specific immunotherapy is a well-recognized effective treatment for allergic asthma triggered by house DMs (HDMs) in children and adults [7,8]. However, about half of the people receiving this allergen-specific immunotherapy treatment have experienced mild side effects, such as a mild rash at the site of the injection, itching and swelling in the mouth, tiredness, headache, sneezing, watery eyes, or mild asthma symptoms [9,10]. Therefore, many patients search for complementary and alternative therapies for improving health in the remission state and non-acute phase of asthma to reduce steroid dosage and prevent acute exacerbation [11,12].

*You-Gui-Wan* (YGW), an herbal remedy, was first mentioned in *Jingyue Quanshu* (1624 AD) and has long been used for enhancing immunity in traditional Chinese medicine (TCM) [13,14]. Previous studies reported that YGW protected the immune function against hydrocortisone-suppressed cytokine expression [14], reduced *Der p*-induced airway hyper-response and remodeling, and alleviated allergen-induced airway inflammation in asthma mouse models [15,16]. YGW also reduced lung inflammation and eosinophil infiltration, induced histone deacetylation in memory T lymphocytes and improved airway inflammation [17]. However, the action mechanism remains for further investigation.

Metabolomics is a systemic, comprehensive and quantitative analysis of changes in global small-molecule metabolites in a biological matrix, which can be directly coupled to a biological phenotype response to a drug treatment or intervention [18]. Because of the complexity of Chinese herbal medicine and the heterogeneity of the etiology of asthma, animals could provide a relative homogenous disease model to explore potential metabolic biomarkers. Therefore, metabolomics has recently attracted much attention to TCM research and is widely used to evaluate its efficacy and clarify the possible action mechanisms [19].

Previous research found gut microbiota associated with metabolic pathways and diseases [20]. The expression of gut microbial-derived metabolites is associated with the IgE response to allergens and asthma [21]. The gut microbiota composition is related to gut metabolism [20,22]. Intestinal gut disorders promote the production of metabolic endotoxins, inflammatory factors, and cytokines etc. [23]. Also, diet and TCM could have a beneficial effect on gut dysbiosis and disordered metabolism [24]. These findings highlight the importance of the gut microbiome in disease development.

In this study, sera and feces from *Der p*-induced asthmatic mice were used for comprehensive metabolomics and fecal microbiota analyses, the associated metabolic pathway and the association between gut microbiome and metabolomics to explore the potential related mechanisms. We aimed to provide molecular evidence for the beneficial effects of YGW in allergic asthma.

## 2. Materials and Methods

### 2.1. Chemicals

All chemicals were obtained from Sigma (St. Louis, MO, USA) unless otherwise specified. An HDM, *D. pteronyssinus* (*Der p*; Catalog no: XPB70D3A2.5; lot no.343206; 2.5 mL/vial) with 4.29 mg protein/vial and 2875 EU endotoxin/vial, was purchased from Greer Laboratories (Lenoir, NC, USA) and was purified from a raw material (lot no. 328219) by bi-level extraction at 1:20 and 1:10 *w*/*v* in 0.125 M ammonium bicarbonate and dialyzed against distilled water.

### 2.2. Herbal Materials

**Figure 1:**
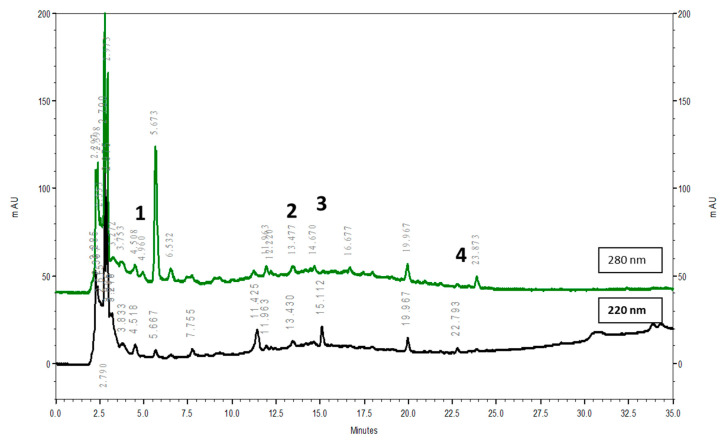
Chromatogram of high-performance liquid chromatography of *You-Gui-Wan* (YGW) decoction. HPLC was run on a Hitachi 5160; 5430 DAD with 5310 oven (40 °C); column: COSMOSIL 5C18-AR-II (250 × 4.6 mm); solvents: A, methanol; B, 0.1% H_3_PO_3_; mobile phase: 0–5 min, 15–20% A; 5–20 min, 20–60% A; 20–30 min, 60–80% A; 30–35 min, 80% A; 35–37 min, 80–15% A; detector: UV: 220 & 280 nm; flow rate: 1 mL/min. 1: gallic acid; 2: caffeic acid; 3: loganin; 4: cinnamaldehyde.

The YGW extract was supplied by Kaiser Pharmaceutics with good manufacturing practices for pharmaceuticals, (Tainan, Taiwan). The composition of YGW included Rehmanniae Radix Preparata (root tuber of *Rehmannia glutinosa*), Dioscoreae Rhizoma (rhizome of *Dioscorea opposita*), Eucommiae Cortex (bark of *Eucommia ulmoides*), Lycii Fructus (fruit of *Lycium chinense*), Corni Fructus (fruit of *Cornus officinalis*), Cuscutae Semen (seed of *Cuscuta australis*), Aconiti Lateralis Radix Preparata (daughter root tuber of *Aconitum carmichaeli*), Cinnamomi Cortex (bark of *Cinnamomum cassia*), Angelicae Sinensis Radix (root of *Angelica sinensis*) and Colla Cornus Cervi (antler of *Cervus elaphus*) at a ratio in the order of 4.4: 2.2: 2.2: 2.2: 2.2: 2.2: 1.1: 1.1: 0.16: 0.16. Each herb was authenticated by Kaiser Pharmaceutics, and the specimen were deposited in Kaiser Pharmaceutics. The chemical profiles of YGW extract were analyzed by HPLC and shown in Figure 1, and four constituents were identified to be gallic acid (**1**), caffeic acid (**2**), loganin (**3**) and cinnamaldehyde (**4**). The concentrated extract was resuspended in distilled water to produce a final concentration of 100 mg/mL for animal administration [16].

### 2.3. Animal Experiment and Statement of Animal Ethics

6- to 8-Week-old male BALB/c mice (20–22 g) were purchased from the National Laboratory Animal Center in Taiwan. Animals were cared and handled according to the Guide for the Care and Use of Laboratory Animals (NIH publication No. 85-12, revised 2010). The animal experiment was reviewed and approved by the Institutional Animal Care and Use Committee of China Medical University (No. 2016-176). 

BALB/c male mice were randomly divided into 5 groups (n = 6 mice each): (1) control (phosphate buffered saline (PBS)); (2) *Der p* only; (3) *Der P* + low-dose YGW (0.2 g/kg); (4) *Der p* + high-dose YGW (0.5 g/kg); (5) *Der p* + dexamethasone (Dex, 1 mg/kg). Dex is a synthetic non-selective glucocorticoid (GC) that is widely used for immunological, allergic and inflammatory diseases treatment [25], as a positive control. The dosage of YGW administrated in this study was based on a physician’s prescription for adults, 2–6 g/70 kg YGW [26,27,28]. 

**Figure 2:**
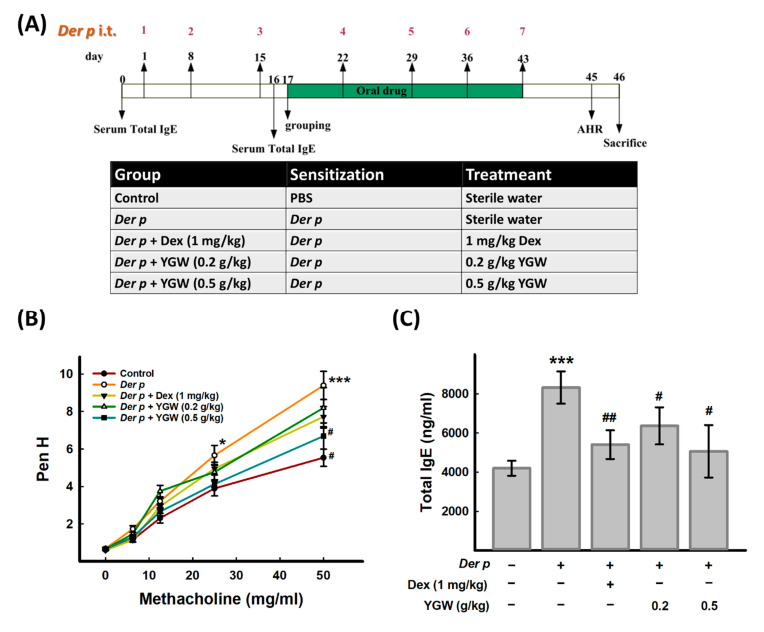
Effect of YGW on airway hyperresponsiveness and serum total IgE in mice. (**A**) A graphical scheme for the timeline of mouse experiments (**B**) Airway hyperresponsiveness was evaluated by methylcholine 2 days after the last tracheal administration of extract of dust mite, *Dermatophagoides pteronyssinus* (*Der p*). (**C**) Serum total IgE level was measured by using a commercial kit. Data mean ± SEM. *** *p* < 0.001, * *p* < 0.05 (vs control); ^##^ *p* < 0.01, ^#^ *p* < 0.05 (vs. *Der p*).

*Der p*-induced allergic asthma in mice mainly followed a previous method [16]. The graphical scheme for the timeline of the mice experiments showed in Figure 2A. Briefly, mice were intratracheally administered 40 μL *Der p* (2.5 μg/μL in PBS) once a week. A total of 7 treatments over 6 consecutive weeks were performed to induce chronic asthma. YGW and Dex were orally administered daily and once a week, respectively. Both YGW and Dex were orally administered 30 min before each *Der p* stimulation. After the last treatment, mice were injected with xylazine (200 μg/mouse) and ketamine (2 mg/mouse) in the abdominal cavity and sacrificed. Blood samples were collected from the brachial artery and serum was obtained after centrifugation and stored at −80 °C for QTOF-MS analysis. After blood collection, mouse feces were collected from the rectum as soon as possible and kept immediately at −80 °C. Due to the collected feces in several animals could not meet the criteria amount. To consider the statistical significance, each group of animals involved 4 mice for metabolomics and gut microbiota analyses.

### 2.4. Measurement of Airway Hyperresponsitivity

The airway resistance of mice was measured by using a single-chamber, whole-body plethysmograph (Buxco Electronics, Troy, NY, USA) with doses of methacholine (Sigma-Aldrich, St. Louis, MO, USA) of 0, 3.125, 6.25, 12.5, 25, and 50 mg/mL. Changes in enhanced pause (Penh) represented airway resistance [15].

### 2.5. Measurement of Total IgE and Tumor Necrosis Factor (TNF)-α Content 

An amount of 100 μL serum was placed in 96-well plates to assess total IgE content with an IgE-specific enzyme-linked immunosorbent assay (ELISA) kit (BD Pharmingen, San Jose, CA, USA), country) [16]. TNF-α concentration was determined with a TNF-α ELISA kit (Boster Biological Technology, Pleasanton, CA, USA).

### 2.6. Serum Metabolomic Profiling

UPLC-QTOF-MS was run on an Agilent 1290 UPLC system (using ACQUITY UPLC HSS T3 column, 2.1 × 100 mm; 1.8 µm; Waters, Milford, MA, USA) coupled with the 6540-Quadrupole-Time-of-Flight (QTOF) mass system (Agilent Technologies, Santa Clara, CA, USA). The LC mobile phase included solvent A (0.1% formic acid in water) and solvent B (acetonitrile, ACN) and was run at a gradient of 0–1.5 min, 2% B; 1.5–9 min, 2 to 50% B; 9–14 min, 50 to 95% B; 14–15 min, 95% B with a flow rate set at 0.3 mL/min. The injection volume was 2 μL. An electrospray ionization (ESI) was used for the ion source, and capillary voltage (40 kV for positive and 35 kV for negative), and fragmenter (120 V). The mass scan range was *m*/*z* = 50–1700. Data processing was performed mainly as Hsu et al. described [29]. MS raw data were converted to an mzXML format by using the Trapper package (Institute for Systems Biology). Then, the mzXML data were processed by using TIPick, an in-house package, to remove the background signals and to detect user-specified metabolites from the MS data. Statistical analysis and interpretation focused on TIPick-identified metabolites only. Scaling-based normalization was performed according to the total ion abundance in the UPLC-MS data.

### 2.7. Bioinformatics Analyses

The involved biological pathway and functional annotation of metabolomics data were analyzed by using the Protein Analysis Through Evolutionary Relationships Classification System (PANTHER), Ingenuity Pathway Analysis (IPA) software (Ingenuity Systems, Mountain View, CA, USA), and MetaboAnalyst 5.0.

### 2.8. Sequencing, Abundance and Diversity Analyses of Gut Microbiota

Total genomic DNA from fecal samples was extracted by column-based method (e.g., QIAamp PowerFecal DNA Kit (Qiagen, Germantown, MD, USA)). A Qubit fluorometer (ThermoFisher Scientific, Waltham, MA, USA) and 16S Metagenomic Sequencing Library Preparation protocol from Illumina were used to assess DNA quality and quantification. Next-generation sequencing was performed as described [30]. Different variable regions of 16S rRNA have been targeted for distinguishing bacteria [31]. The V3-V4 region was identified for distinguishing intestinal bacteria species and was amplified by using a specific primer with a barcode [31]. Reads were quality filtered by using Quantitative Insights Into Microbial Ecology (QIIME) [32], and chimeric sequences were removed by UCHIME [33]. The processed sequencing reads (effective tags) were clustered into operational taxonomic units (OTUs) at 97% sequence identity by using UPARSE [34]. Then, diversity analysis was performed, and the taxonomy classification of OTUs was assigned according to information retrieved from the SILVA Database v.132 [35,36]. Differences in bacteria abundance were calculated by linear discriminant analysis (LDA) effect size (LEfSe) [37] and OTU abundance information was normalized by a standard sequence number corresponding to the sample with the least sequences. Differences in abundance between groups were tested by multiple comparison adjustments [38], and subsequent analysis of alpha and beta diversity involved these output normalized data.

### 2.9. Statistical Analysis

Multivariate statistical analysis of YGW metabolomic data, including principal component analysis (PCA) and partial least-squares discriminant analysis (PLS-DA), was used to analyze the covariance between the measured peak intensities in the MS spectra and the response variables [29]. All analyses involved using IBM SPSS v23.0. Descriptive statistics are presented as mean ± SEM, median (range), or number (%). Student *t*, Fisher exact, or chi-square test was used to compare groups. Paired *t*-test or Wilcoxon signed-rank test was used to compare paired data. All calculated *p*-values were two-tailed. Statistical significance was defined at *p* < 0.05.

## 3. Results

### 3.1. Effect of YGW on the Airway Hyperresponsivity and Total IgE Level in Der p-Induced Mouse Allergic Asthma

The allergic asthma model was established by intratracheally repetitive administration of *Der p* stimulus to mouse airways as previously described [15,16], which resulted in mice with allergic asthma exhibiting airway hyperresponsivity and high total IgE levels, similar to those of clinical symptoms of asthma patients [39]. The disease model group (*Der p*) showed significantly increased Penh value at both 25 and 50 mg/mL methacholine as compared with the control group, which was significantly decreased by high-dose YGW treatment (*Der p* + YGW (0.5 g/kg) (Figure 2B). Furthermore, the positive group (*Der p* + Dex) showed a similar effect as the low-dose YGW group. Therefore, the high-dose YGW group was used for the following metabolomics and gut microbiota studies.

Total serum IgE level is higher in most patients with asthma than people without asthma [40], as well as those mice in HDM-treated than control [39]. To understand whether YGW regulates humoral immunity, total IgE content in mouse serum was measured. Total serum IgE level was higher in only *Der p*-treated group than controls and was effectively reduced by YGW administration (Figure 2C).

### 3.2. Metabolomics Profiling of YGW Treatment in Der p-Induced Allergic Asthma in Mice

Serum metabolomic profiling by LC-MS was first analyzed by multivariate statistical analysis (PCA and PLS-DA) to discern the differences among control, *Der p,* and treatment groups (*Der p* + YGW, 0.5 g/kg). Exploratory PCA was used to detect intrinsic clustering and possible outliers in the metabolome. The plot of principal component 1 versus 2 scores (Supplementary Figure S1A) (R^2^X = 0.900, Q^2^ = 0.729) showed an obvious separation among control, *Der p,* and YGW treatment groups. By further applying PLS-DA, we observed a reasonably good separation between *Der p* and control groups (R^2^X = 0.896, R^2^Y = 0.661, Q^2^ = 0.349) (Supplementary Figure S1B). Moreover, findings for the YGW treatment group were closer to those of the control group, which suggests that YGW had a therapeutic effect on *Der p*-induced mouse allergic asthma. These results also revealed that the models were suitable for predicting the variables that contributed to the class clustering and had a low risk of overfitting. 

Unpaired *t*-test was used to choose metabolites with statistically significant change in value (*p* < 0.05, control vs *Der p*) (Table 1). Then, we used variable importance in projection (VIP) analysis to determine the metabolites that contributed most to the model. The metabolites with VIP scores > 1.0 were selected. Significantly *Der p*-altered biosignatures included 10 metabolites—acetylcarnitine, carnitine, hypoxanthine, tryptophan, phenylalanine, norleucine, isoleucine, betaine, methionine, and valine (Table 1). Seven bio-signatures could be significantly reversed by YGW treatment. Values for the remaining 3 metabolites—carnitine, hypoxanthine, and phenylalanine—could also be reversed by YGW treatment but without statistical significance (Table 1).

### 3.3. Effect of YGW on Metabolic Pathways 

**Figure 3:**
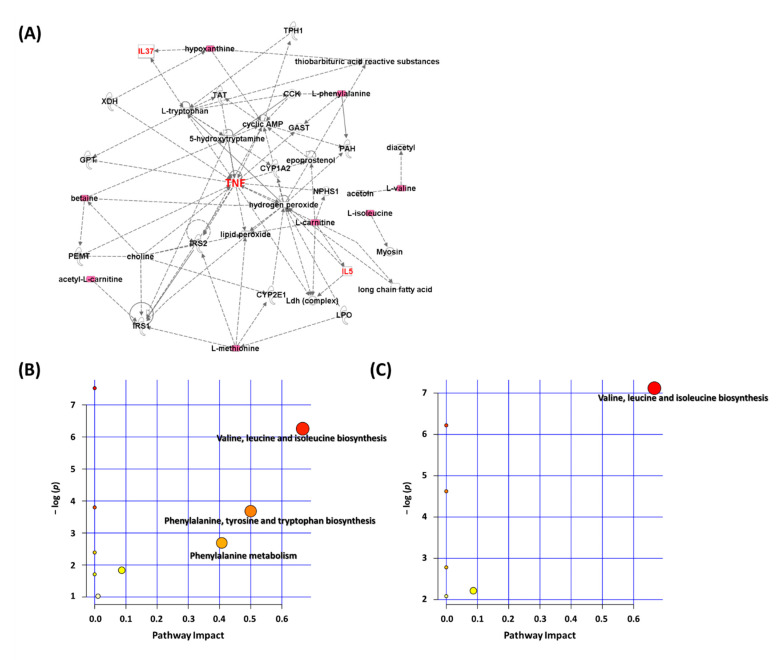
Metabolite signatures analysis of pathways affected by *Der p*-induced chronic asthma and YGW treatment in mice. (**A**) Network pathways were identified by IPA and MetaboAnalyst software. Molecular network of the *Der p* group, direct interactions are represented by continuous lines and indirect interactions by dashed lines. The red nodes represent upregulated metabolites and downregulated metabolites by green nodes. Metabolites were inferred in the *Der p* group from changes in serum levels of intermediates during substance metabolism. Pathways were affected by (**B**) *Der p* and (**C**) YGW treatment (0.5 g/kg).

Biochemical pathways responsible for the observed metabolic abnormalities were further identified by IPA. In the network analysis, *Der p*-induced serum metabolites related to allergic asthma tended to gather in a single network (Figure 3A). TNF-α was the most important marker among the several "hub" molecules at the center of this network. The TNF-α level was higher in *Der p*-induced allergy in mice and was significantly reduced by Dex and YGW (0.5 g/kg) treatments (Supplementary Figure S2).

To further evaluate the underlying implication of the metabolites with changed content, MetaboAnalyst 5.0 was used to analyze the metabolic pathways. To define the relationships among the metabolites, we used pathway analysis of the 10 potential biomarkers by using “mouse” as the specific model organism and revealed 7 pathways as the most important in *Der p*-induced allergic asthma. Seven metabolites with values significantly reversed by YGW were used to detect the potential pathways. The most important metabolic pathways (pathway impact > 0.1 and *p* < 0.05) were valine, leucine, and isoleucine biosynthesis (branched-chain amino acid [BCAA] metabolism); phenylalanine, tyrosine, and tryptophan biosynthesis (aromatic amino acid [AAA] biosynthesis); and phenylalanine metabolism (Figure 3B). In Figure 3C, the results suggested that valine, leucine, and isoleucine biosynthesis (BCAA metabolism) chiefly contributed to the pharmacological effects of YGW in *Der p*-induced allergic asthma.

### 3.4. Effect of YGW on Gut Microbiota in Der p-Induced Allergic Asthma in Mice

**Figure 4:**
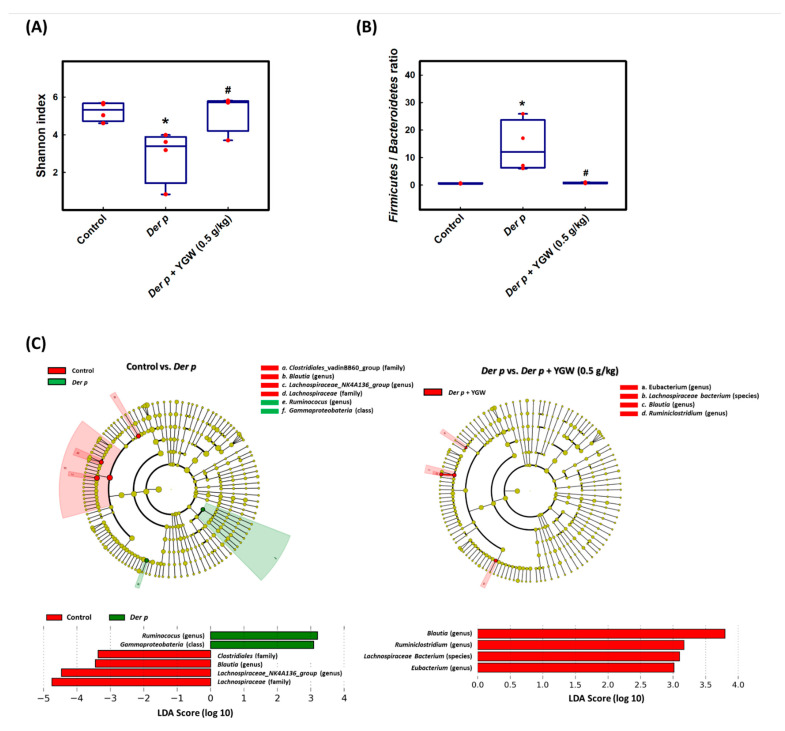
Summary of gut microbiota affected by *Der p*-induced asthma and YGW treatment in mice. (**A**) Gut microbiota indicated by Shannon diversity index compared among control, *Der p*, and *Der p* + YGW (0.5 g/kg) groups. (**B**) Ratios of *Firmicutes*/*Bacteroidetes* are relative abundance. (**C**) Cladogram generated from LEfSe analysis shows the association between taxon (the levels represent, from inner to outer rings, phylum, class, order, family, and genus). Data are mean ± SEM. * *p* < 0.05 (vs control); # *p* < 0.05 (vs Der p).

Multiple studies have shown that amino acid metabolic disorder is related to gut microbiota [41,42,43]. To assess the structural changes of fecal microbial communities with control, *Der p,* and YGW treatment, we generated an average of more than 54,000 sequences from all samples and retained 509 OTUs, then analyzed the diversity of the microbial community (alpha diversity). The average microbiota diversity was slightly lower with *Der p* only than the control, but the lower microbiota diversity in the *Der p* group was ameliorated by YGW treatment (Figure 4A).

**Figure 5:**
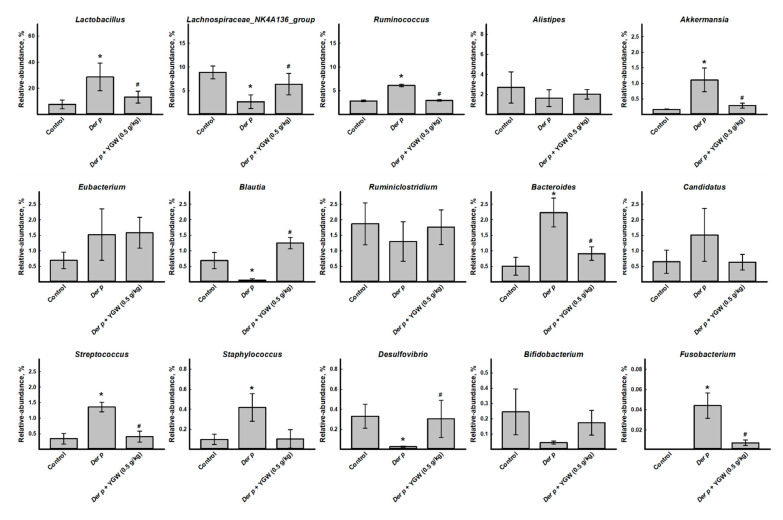
Relative abundance of the top 15 gut microbiota genera in control, *Der p* and *Der p*+ YGW (0.5 g/kg) groups. Differential expression of genera of gut microbiota. Shows relative abundance of *Lactobacillus*, *Lachnospiraceae*, *Ruminococcaceae*, *Alistipes*, *Akkermansia*, *Eubacterium*, *Blautia*, *Ruminiclostridium*, *Bacteroides*, *Candidatus*, *Streptococcus*, *Staphylococcus*, *Desulfovibrio*, *Bifidobacterium*, and *Fusobacterium*. Data are mean ± SEM. * *p* < 0.05 (vs control); ^#^ *p* < 0.05 (vs *Der p*).

Furthermore, to intuitively assess the specific changes in the microbial community in gut microbiota between groups, we analyzed the relative abundance of the dominant taxa identified by sequencing in each group. The top 15 taxa were used to generate the relative abundance superposition histogram at the genus level (Figure 5). Among the top 15 taxa (Figure 5), the *Der p* group showed an increased relative abundance of *Lactobacillus*, *Ruminococcus*, *Akkermansia*, *Eubacterium*, *Bacteroides*, *Candidatus*, *Streptococcus*, *Staphylococcus*, and *Fusobacterium* but decreased abundance of *Lachnospiraceae_NK4A136_group*, *Alistipes*, *Ruminiclostridium*, *Blautia*, *Desulfovibrio*, and *Bifidobacterium*. YGW treatment partially reversed the change in abundance (Figure 5). LEfSe analysis, a biomarker discovery tool for high-dimensional data, was used to explore the group differences by analysis of taxon abundance in the gut microbiota (from phylum to species). LDA revealed distinct taxa in the gut microbiota of the groups. A cladogram was generated by LEfSe analysis of sequences from each sample. The top nine differences (LDA score > 3.0) in intestinal flora between control and *Der p*-only groups and between *Der p* and *Der p*+ YGW groups are in Figure 4C. LEfSe analysis revealed that the family *Clostridiales*_*vadinBB60*_group, and *Blautia* and *Lachnospiraceae_NK4A136*_group were enriched in the control group; *Ruminococus* was enriched in the *Der p* group; and *Eubacterium*, *Blautia*, *Ruminiclostridium*, and *Lachnospiraceae_NK4A136*_group were enriched in the *Der p*+ YGW group. The composition of gut microbiota in different groups was profoundly altered by *Der p*-induced allergic asthma in mice.

Previous studies revealed a high relative abundance of *Firmicutes/Bacteroidetes* in the gut microbiota of asthma patients [44]. Thus, we detected whether the ratio of *Firmicutes* to *Bacteroidetes* abundance was increased in the *Der p*-induced group. The ratio of *Firmicutes* to *Bacteroidetes* abundance in control, *Der p*, and *Der p*+ YGW groups was 0.59, 11.49, and 0.70, respectively (Figure 4B). *Der p* significantly enhanced the ratio, and YGW treatment reversed the ratio, but without significant difference between the control and *Der p*+ YGW group.

### 3.5. Correlation between Metabolomic Signatures and Microbial Community

**Figure 6:**
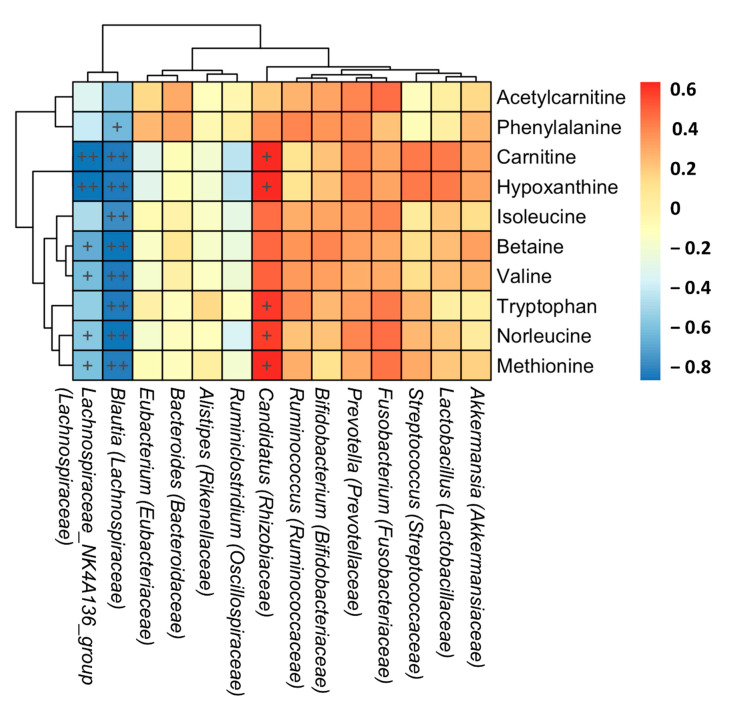
Heat map of correlations between the level of metabolites and microbiota composition. The heat map shows Spearman’s correlations between significantly changed genera of gut microbiota and selected serum metabolites between the control and *Der p* group. Color intensity represents the magnitude of correlation. Red represents positive correlations and blue negative correlations. + *p* < 0.05; ++ *p* < 0.01.

To investigate the interrelations between the change in metabolite levels and microbiota composition, we used Spearman rank correlation analysis of genera with the highest expression in gut microbiota and levels of the selected serum metabolites between the control and *Der p* groups, then represented these in a heat map (Figure 6). Multiple correlation analysis showed a positive correlation (*p* < 0.05) between *Candidatus* and carnitine (r = 0.636), hypoxanthine (r = 0.636), norleucine (r = 0.587), methionine (r = 0.643), and tryptophan (r = 0.601). However, *Blautia* and *Lachnospiraceae_NK4A136_group* showed a negative correlation (*p* < 0.05) with most of the 10 selected metabolites: acetylcarnitine, carnitine, hypoxanthine, phenylalanine, norleucine, isoleucine, betaine, methionine, and valine. Therefore, the expression of the 10 selected metabolites was well correlated with gut microbiota composition. 

## 4. Discussion

This study showed *Der p*-induced airway hypersensitivity and inflammation in mice, which could be alleviated by YGW treatment, as shown in a previous study [16]. Serum metabolomic analysis showed that *Der p* markedly elevated the levels of 10 metabolites—acetylcarnitine, carnitine, hypoxanthine, tryptophan, phenylalanine, norleucine, isoleucine, betaine, methionine, and valine. These metabolites are mainly related to BCAA metabolism, AAA biosynthesis, and phenylalanine metabolism. YGW administration reversed the levels of 7 of the 10 metabolites, chiefly BCAA metabolites. 16S DNA sequencing revealed that YGW profoundly changed the composition of *Der p*-induced gut microbiota, and multiple correlation analyses indicated a good correlation between gut microbiota composition and levels of the 10 selected metabolites.

Previous studies also demonstrated that DMs could directly activate innate immune cells, and Type I IgE-mediated hypersensitivity reactions and inflammation [45]. Repeated DM challenge in mice resulted in chronic inflammation characterized by increased number of lymphocytes and eosinophils in bronchoalveolar lavage fluid [39], airway hypersensitivity and inflammatory cell infiltration [15], which are similar to our results. The Penh method for measuring airway resistance is somewhat controversial [46,47]. It is still the main measurement used in most studies (~49%) examining lung function in animal models in the past years [48].

Metabolomics is a comprehensive characterization of metabolites in biological systems that generates unique chemical fingerprints for specific cellular processes. In particular, metabolomics is increasingly used to diagnose disease, understand disease mechanisms, identify novel drug targets, and monitor therapeutic outcomes, etc. [49]. Asthma and airway inflammation are complex and respond to infectious stimuli, which may result in a broad spectrum of possible metabolic products [42]. Metabolomics might provide unique and novel insights into asthma. In this study, *Der p* induced a mouse metabolic profile with 10 metabolic signatures—acetylcarnitine, carnitine, hypoxanthine, tryptophan, phenylalanine, norleucine, isoleucine, betaine, methionine, and valine, significantly changed, which could be potential biomarkers for *Der p*-induced allergic asthma. Tryptophan, phenylalanine, isoleucine, methionine, and valine belong to essential amino acids, which cannot be synthesized by mammalian cells. Gut microbiota such as the genera of *Bacteroides*, *Clostridium*, *Candidatus*, *Propionibacterium*, *Fusobacterium*, *Streptococcus*, *Lactobacillus*, *Akkermansia*, and *Bifidobacterium* were associated with the elevated levels of serum methionine, BCAAs (isoleucine, leucine, and valine), and AAAs (tryptophan, phenylalanine, and tyrosine) [50,51]. Intestinal microbes can provide amino acids to meet host requirements, contributing to the energy delivery and modulating amino acid homeostasis [51,52]. A recent study also showed that HDM could cause immune system disorders correlated with reduced diversity of gut microbiota [53]. In addition, gut microbiota genera such as *Staphylococcus*, *Rumminococcaceae*, *Lachnospiraceae_NK4A136*_group, and *Streptococcus* have been implicated in the predisposition to allergy, airway inflammation, or asthma in both experimental models and clinical studies [54]. A reduction in *Bacteroidetes* and *Bifidobacteria* content has been associated with asthma [55]. Our study revealed that the content of some microbiota genera, including *Lachnospiraceae_NK4A136*_group, *Bacteroidetes, Blautia*, and *Desuflovibrio*, etc., was markedly reduced and some increased in *Der p*-induced allergic asthma in mice. After YGW treatment, the intestinal flora structure tended to be reversed to similar to that of the control group. Also, correlation analysis of the level of metabolites and gut microbiota demonstrated a positive correlation between the content of some genera and the changed levels of some amino acids in *Der p*-induced mouse allergic asthma. This study provides support for the correlation between gut microbiota composition and *Der p*-induced allergic asthma.

Moreover, a previous study showed the level of acetylcarnitine and valine significantly increased in an ovalbumin (OVA)-induced asthma model [56], which is consistent with our results. Additionally, Ho et al. recently reported increased tryptophan level in HDM-induced allergic asthma [57], and high phenylalanine level was also detected in asthma patients [58]. Furthermore, norleucine, isoleucine, betaine, and methionine found in this study have never been previously found related to asthma. However, carnitine expression in asthma is controversial, some studies showing high carnitine levels in asthma patients [58,59,60], but others showing decreased plasma carnitine levels in asthma [61,62].

Cytokines/chemokines promote immune cell recruitment and activation, which play an integral role in airway inflammation. Many metabolites with altered expression share manifold statistical associations to inflammatory cells and cytokines, so they may be biologically relevant metabolic changes linked to airway inflammation [63]. For instance, hypoxanthine level is associated with multiple inflammatory markers including neutrophil count and the cytokines IL-4, IL-5, IL-6, IL-8, IL-37, TNF-α, and IL-1β [63]. The present investigation found *Der p*-induced metabolic profile changes, involved BCAA metabolism, AAA biosynthesis, and phenylalanine metabolism. Increased TNF-α level generates inflammatory responses, induces lipolysis, and increases phenylalanine flux [64]. Likewise, some pathogens can upregulate interferon, which could in turn upregulate tryptophanyl tRNA synthetase and be involved in tryptophan catabolism [65]. Furthermore, BCAAs can affect protein synthesis and decomposition and promote glutamine synthesis. BCAAs mediate proteins, DNA, and RNA syntheses in lymphocytes and regulate immune functions [66,67] as well as affect allergic responses to food and allergy-related outcomes [68]. The amount of tryptophan that contributes to protein synthesis or serotonin production is minimal because more than 95% is converted into kynurenine and its related molecules, initially by indoleamine 2,3-dioxygenase (IDO), which metabolizes tryptophan to produce kynurenine derivatives in antigen-presenting cells and other cells resident in lymph nodes and inflammatory tissue [69,70]. The expression of IDO is induced by IFN-γ and inhibited by Th2 cytokines including IL-4 and IL-13. Also, people with asthma show reduced IDO activity [71,72,73]. A recent study showed that tryptophan and kynurenine levels were higher and lower IgE and IDO activities in people with asthma and allergic rhinitis [71,72]. These results were similar to ours.

Malkawi et al. showed that Dex could induce perturbation in several pathways, such as amino acid metabolism, pyrimidine metabolism, and nitrogen metabolism [74]. Furthermore, Dex treatment significantly elevates serum levels of tyrosine and hydroxyproline and significantly reduces those of in phenylalanine, lysine, and arginine. Dex treatment could change the diversity and relative abundance of intestinal flora in OVA-induced asthma rats [75]. Accumulating evidence indicates that YGW can modulate immune disorders, especially boosting the immune function to strengthen the line of defense against pathogens [11]. A previous study showed that YGW could attenuate Der p-induced inflammation by downregulating TGF-β, IL-4, IL-5, IL-13, and inhibiting NF-κB activation [16]. In this study, we further demonstrated that YGW could ameliorate *Der p*-induced gut dysbiosis and significantly reverse the increased level of 7 metabolites—acetylcarnitine, tryptophan, norleucine, isoleucine, betaine, methionine, and valine and chiefly affect valine, leucine, and isoleucine biosynthesis. These results were similar to the Dex-treated group. Moreover, levels of the top 10 high-impact metabolites showed a good correlation with microbial community profiling. Hence, YGW apparently ameliorates *Der p*-induced allergic asthma.

The results and interpretation of this study possess the following limitations: (1) the metabolite panel used in our study contained the selected metabolites via LC/MS only and might not cover the whole metabolome and (2) the number of animals recruited was relatively small, and our findings cannot be fully extrapolated to human asthma patients. Therefore, further validating the observed biosignatures from *Der p*-induced mouse allergic asthma in human asthma patients is necessary.

## 5. Conclusions

In summary, *Der p* chiefly disturbed amino acid metabolism and imbalance in intestinal flora in mice with allergic asthma. YGW treatment could mainly improve BCAA metabolism and ameliorate gut microbiota disorder. Our study provides scientific evidence for YGW administration with potential benefits for allergic asthma by ameliorating gut dysbiosis and improving the metabolome imbalance.

## Figures and Tables

**Table 1:** Metabolites with significantly changed levels between control and model group.

Metabolite Name	HMDB	*Der p*			*Der p* + YGW (0.5 g/kg)	
		*Der p*/Control	*p* Value (vs. Control)	VIP Value (vs. Control)	*Der p* + YGW/Control	*p* Value (vs. *Der p*)
Acetylcarnitine ^a^	HMDB0000201	1.432	0.033	4.829	0.967	0.030
Betaine ^a^	HMDB0000043	1.242	0.010	3.241	0.915	0.019
Carnitine ^a^	HMDB0000062	1.212	0.028	1.599	1.056	0.244
Hypoxanthine ^a^	HMDB0000157	1.212	0.028	1.595	1.056	0.246
Isoleucine ^a^	HMDB0000172	1.227	0.014	3.906	0.902	0.007
Methionine ^a^	HMDB0000696	1.289	0.041	1.430	0.999	0.037
Norleucine ^a^	HMDB0001645	1.238	0.021	1.666	0.907	0.018
Phenylalanine ^a^	HMDB0000159	1.230	0.031	2.711	0.998	0.127
Tryptophan ^a^	HMDB0000929	1.346	0.049	1.577	0.875	0.048
Valine ^a^	HMDB0000883	1.192	0.031	3.513	0.896	0.029
2-Methylbutyryl-L-carnitine	HMDB0000378	1.679	0.031	0.049	1.186	0.233
Valeryl-L-carnitine	HMDB0013128	1.679	0.031	0.049	1.186	0.367
L-3-Phenyllactic acid	HMDB0000748	1.504	0.014	0.013	1.053	0.012
Ketoleucine	HMDB0000695	1.484	0.027	0.065	1.043	0.016
Tetradecanoyl-L-carnitine	HMDB0005066	1.405	0.027	0.051	0.937	0.025
3-Hydroxybutyric acid	HMDB0000357	1.401	0.024	0.113	0.930	0.017
D-*threo*-Isocitric acid	HMDB0001874	1.358	0.026	0.112	1.479	0.279
2-Hydroxybutyric acid	HMDB0000008	1.314	0.028	0.069	1.128	0.112
Citric acid	HMDB0000094	1.221	0.026	0.066	1.275	0.745
Pantethine	HMDB0003828	1.150	0.018	0.011	0.958	0.057
Hydroxyphenyllactic acid	HMDB0000755	0.663	0.005	0.017	0.856	0.053
Phosphorylcholine	HMDB0001565	0.616	0.035	0.009	0.879	0.179
Lysine	HMDB0003405	0.217	0.010	0.936	1.018	0.002

VIP: variables important for the projection. ^a^ Potential biomarker candidates in *Der p*-altered metabolite signatures.

## Data Availability

The data presented in this study are available on reasonable request from the corresponding author.

## Supplementary Materials

The following are available online at https://www.mdpi.com/article/10.3390/biom11060812/s1, Figure S1: PCA and PLS-DA analysis of metabolites with changed expression among control, *Der p*, and *Der p* + YGW (0.5 g/kg) groups, Figure S2: Effect of YGW on TNF-α level in mouse serum.

## Abbreviations

amino acid	AA
aromatic amino acid	AAA
branch-chain amino acid	BCAA
Chronic obstructive pulmonary disease	COPD
*Dermatophagoides pteronyssinus*	*Der p*
*Dermatophagoides farina*	*Der f*
dexamethasone	dex
house dust mites	HDM
Ingenuity Pathway Analysis	IPA
linear discriminant analysis	LDA
linear discriminant analysis effect size	LEfSe
operational taxonomic units	OTUs
partial least-squares discriminant analysis	PLS-DA
principal component analysis	PCA
Protein Analysis Through Evolutionary Relationships Classification System	PANTHER
Quantitative Insights Into Microbial Ecology v2	QIIME2
traditional Chinese medicine	TCM
variables important for the projection	VIP
*You-Gui-Wan*	YGW
