# Supplementary material for: Effect of You-Gui-Wan on House Dust Mite-Induced Mouse Allergic Asthma via Regulating Amino Acid Metabolic Disorder and Gut Dysbiosis
Source: Biomolecules. 2021 May 30;11(6):812. doi: 10.3390/biom11060812 (PMC8229888; doi:10.3390/biom11060812)
Supplement: Supplementary file 1 [file biomolecules-11-00812-s001.zip › biomolecules-1192808-final-suppl-1.pdf]

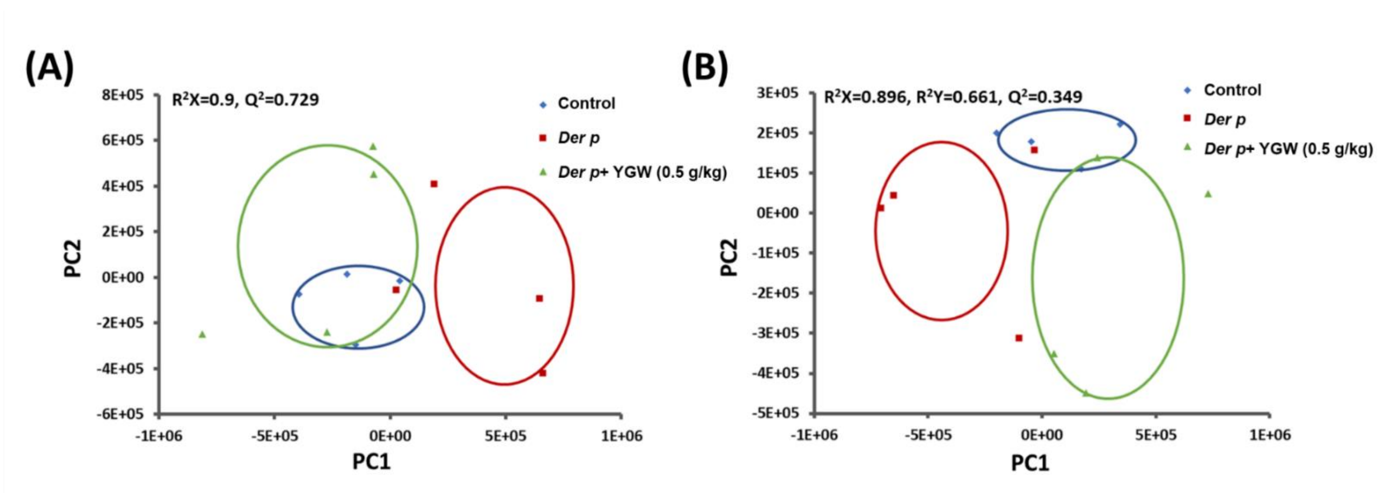

Supplementary Figure S1. PCA and PLS-DA analysis of metabolites with changed expression among control, *Der p* and *Der p* + YGW (0.5 g/kg) groups. Scatter plots of scores of (A) PCA and (B) PLS-DA by LC-QTOF-MS of serum from control (blue), *Der p* (red), *Der p*+ YGW (0.5 g/kg) groups (green).

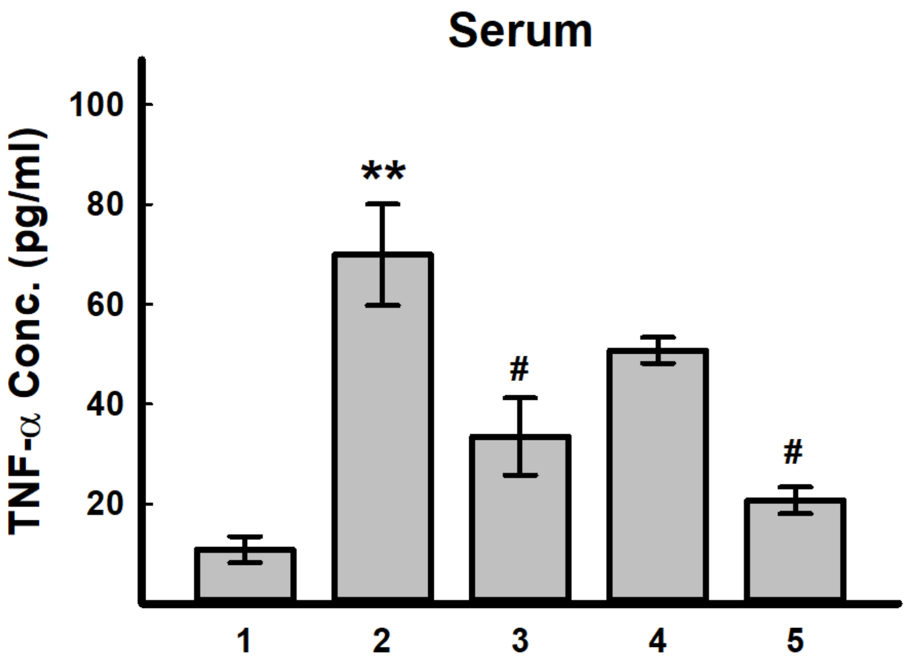

Supplementary Figure S2. Effect of YGW on TNF-α level in mouse serum. Data are mean ± SEM. *** $p < 0.001$, * $p < 0.05$ (vs control); ## $p < 0.01$, # $p < 0.05$ (vs Der p). **1**: control, **2**: *Der p*, **3**: *Der p* + dex, **4**: *Der p* + YGW (0.2 g/kg), **5**: *Der p*+ YGW (0.5 g/kg).
